# Dr. Edward Enos Hopkins

**Published:** 1913-12

**Authors:** 


Dr. Edward Enos Hopkins, Buffalo, 1908, formerly of Rochester, died at Honeoye Falls, October 24, aged 31.

